# Gastric Scintigraphy as a Bridge Between Objective and Clinical Evaluation of Delayed Gastric Emptying Following Pancreatic Surgery—A Pilot Study

**DOI:** 10.3390/diseases14080287

**Published:** 2026-08-10

**Authors:** Artur Rebelo, Sara Seyedinia, Nepomuk Sochor, Jessica Döbereiner, Matthias Sommerer, Ulrich Ronellenfitsch, Johannes Klose, Andreas Odparlik, Alexander Heinzel, Jörg Kleeff

**Affiliations:** 1 Department of Visceral, Vascular and Endocrine Surgery, University Hospital Halle (Saale), Martin-Luther-University Halle-Wittenberg, Ernst-Grube-Street 40, 06120 Halle (Saale), Germanyulrich.ronellenfitsch@uk-halle.de (U.R.); joerg.kleeff@uk-halle.de (J.K.); 2 Department of Nuclear Medicine, University Hospital Halle (Saale), Martin-Luther-University Halle-Wittenberg, 06120 Halle (Saale), Germany

**Keywords:** delayed gastric emptying, gastric scintigraphy, pancreatoduodenectomy, gastroparesis, postoperative complications, pancreatic surgery, gastric emptying study

## Abstract

Introduction: Delayed gastric emptying (DGE) is a common complication after pancreatoduodenectomy, occurring in up to two-thirds of patients. Although rarely life-threatening, DGE prolongs hospitalization and increases healthcare costs. The International Study Group of Pancreatic Surgery (ISGPS) definition enables retrospective grading but lacks objective physiological assessment. This pilot study evaluated the feasibility and clinical relevance of standardized postoperative gastric scintigraphy. Methods: Six patients undergoing pancreatoduodenectomy between January and May 2025 received one or two standardized liquid gastric emptying scintigraphies on postoperative days (POD) 4–6 and 10–13. Quantitative analysis included half-emptying time (T½) and residual activity (RA%) at 30 and 60 min. Findings were correlated with ISGPS-defined DGE. Results: Eleven scintigraphic examinations were performed. Clinically relevant ISGPS Grade B/C DGE occurred in two patients and was associated with persistently elevated RA% at 60 min and prolonged T½ on early scintigraphy. Two additional patients showed scintigraphic abnormalities without meeting clinical DGE criteria, without confirmed outcome-level consequences. Conclusions: Standardized gastric scintigraphy may provide an objective and reproducible method for evaluating DGE after pancreatoduodenectomy and may complement clinically based ISGPS definitions.

## 1. Introduction

Pancreatoduodenectomy is the standard surgical treatment for pancreatic head diseases. Postoperative complications include pancreatic fistula, hemorrhage, and delayed gastric emptying (DGE) occurring in up to two-thirds of patients even in high-volume centers. DGE as one the main complications, prolongs duration of hospital stay, delays discharge, and is associated with increased healthcare costs. The International Study Group of Pancreatic Surgery (ISGPS) defines DGE as the inability to tolerate normal oral intake by the end of the first postoperative week, often requiring nasogastric tube placement, and is classified into grades A–C according to duration and intervention requirements [1,2].

While the definition allows for retrospective assessment of DGE, it does not guide the diagnosis or management of patients with impaired gastric emptying, nor does it enable quantification in clinical trials, selective enrollment of patients for specific interventions, or objective measurement of the severity of gastroparesis. Furthermore, a confirmed diagnosis of gastroparesis requires measurement of delayed gastric emptying via an appropriate test, such as gastric scintigraphy or ^3^C-octanoate or ^13^C-acetate breath [3].

A recent meta-analysis evaluated strategies to reduce DGE after pancreatoduodenectomy, including pylorus resection versus preservation, enhanced recovery after surgery (ERAS), epidural anesthesia, and nasojejunal or jejunostomy feeding. The analysis of 5930 patients from 29 studies suggested that pylorus resection, adherence to ERAS protocols, and omission of jejunostomy/nasojejunal feeding were associated with lower rates of DGE. However, the evidence is limited by heterogeneous definitions of DGE, the absence of standardized objective measurement methods, and reliance on observational studies, highlighting the need for more precise and quantifiable approaches in future research [4].

Another systematic review identified several objective methods to assess DGE after pancreaticoduodenectomy, including gastric scintigraphy, acetaminophen absorption tests, fluoroscopy, and the ^13^C-acetate breath test. For gastric scintigraphy, there is some evidence supporting correlation with DGE, though overall use is limited. Previously published studies had the limitation of highly variable protocols and inconsistent correlation with the ISGPS clinical definition of DGE, highlighting the need for a standardized, real-time, and objective modality to evaluate gastric emptying after pancreaticoduodenectomy [5,6,7].

In this manuscript, we report on the pilot use of gastric emptying scintigraphy to assess DGE, aiming to guide diagnosis and management, enable objective quantification in future clinical trials, facilitate selective enrollment for interventions, and provide a reproducible measure of gastroparesis severity.

## 2. Methods

### 2.1. Patient Sample

For this pilot study, we retrospectively selected the patients who underwent pancreatoduodenectomy at our institution and received gastric scintigraphy between January and May 2025. Our institution performs 60–80 pancreatoduodenectomies annually; accordingly, approximately 25–33 patients underwent pancreatoduodenectomy during the 5-month inclusion period. Of these, 6 patients (approximately one-fifth) underwent postoperative gastric scintigraphy. Patients were not enrolled consecutively, and no formal predefined inclusion or exclusion criteria beyond clinical indication were applied; selection was made at the discretion of the treating surgical team, which we acknowledge introduces selection bias (see Limitations). Scintigraphy was performed in patients with clinical suspicion of delayed gastric emptying, defined as inability to advance to a solid oral diet by postoperative day 3, recurrent vomiting or high nasogastric output, or need for nasogastric tube reinsertion, as judged by the treating surgical team. The Ethics Committee of the Medical Faculty of Martin Luther University Halle-Wittenberg approved the present study (2025-185). Informed consent was obtained from all patients prior to the performance of gastric scintigraphy.

Demographic and clinical data were collected for each patient, including age (years), sex (male/female), height (m), body weight (kg), diagnosis, and treatment. Preoperative clinical status was assessed using the Eastern Cooperative Oncology Group (ECOG) performance status and American Society of Anesthesiologists (ASA) physical status classification. Medical history variables included diabetes mellitus and smoking status. Clinical parameters recorded preoperatively included presence of jaundice and preoperative biliary drainage.

### 2.2. Surgery

Surgery was performed using highly standardized procedures for pancreatoduodenectomy at our institution. All cases were discussed in the multidisciplinary tumor board, and operations were performed by two experienced pancreatic surgeons. Intraoperatively, a naso-gastric tube was placed and removed in the operating theater at the end of the procedure. Patients were routinely transferred to the intensive care unit after surgery, with rapid extubation and supportive care as indicated. Following an uneventful initial course, patients were moved to the intermediate care unit, and oral intake was initiated according to clinical recovery. Physiotherapeutic exercises were started on the first postoperative day.

Intraoperative data included the type of surgical procedure, whether vascular resection was performed and its specific type, and operation time (minutes). Pathological assessment recorded tumor location, histology, pathological or clinical tumor stage ((y)pT) and nodal stage ((y)pN) according to the TNM 8th edition, number of lymph nodes positive and examined, presence of distant metastasis ((y)cM), tumor grade (G), resection margin status (R), perineural invasion, lymphovascular invasion, and microvascular invasion. Postoperative outcomes included overall complications, severe complications graded ≥3 according to Clavien-Dindo, postoperative pancreatic fistulas (POPF), biliary fistulas, abdominal fluid collections, DGE, postpancreatectomy hemorrhage (PPH), sepsis, pulmonary complications, length of hospital stay and ICU stay, reoperations, in-hospital mortality and postoperative surgical site infections during hospitalization.

### 2.3. Scintigraphy

We performed two liquid gastric emptying scintigraphies at two different postoperative time points: between days 4 and 6, and 11 and 13. Patients fasted for a minimum of 4 h prior to each examination. To minimize factors affecting gastric emptying, prokinetic agents, opioids, anticholinergics, and laxatives were withheld for at least 48 h before the study. Patients were also instructed to refrain from smoking on the day of imaging.

All studies were performed on a morning time schedule and blood glucose levels were confirmed to be below 200 mg/dL prior to imaging. Each examination utilized a radiolabeled liquid meal consisting of 18.5–37 MBq technetium-99m diethylenetriamine pentaacetic acid (^99m^Tc-DTPA) added to 300 mL of water, followed by an additional swallow of a glass of water to clear the residual activity from the esophagus.

Imaging was performed using a gamma camera equipped with a general-purpose collimator (Siemens Healthineers, Erlangen, Germany) and a 128 × 128 matrix to optimize count statistics. Image acquisition was performed in supine position. Immediately after ingestion of the liquid meal, a static image encompassing the abdominal region was acquired for one minute (early image). Directly afterwards dynamic imaging was performed with one frame acquired per minute for 30 min. This was followed by delayed static images obtained at 60, 90 and/or 110 min.

Regions of interests (ROIs) included the entire gastrointestinal activity as well as a separated ROI encompassing the stomach were drawn on early anterior projection images. On delayed images, ROIs were drawn only over the region of gastric activity. The following parameters were derived: time-activity curve, half-emptying time (T½), percentage of the residual gastric activity (RA%) at 30, and 60 min.

Based on the study of Dhukia et al. 2024, we considered a T1/2 of 29 min ±19 and RA% of 39 ± 18% at 30 min and 15 ± 16% at 1 h, respectively, as normal in this study. Values greater than these limits were classified as delayed gastric emptying [8]. As the study parameters were occasionally discordant (e.g., an isolated abnormal T½ with normal RA%), the overall scintigraphic diagnosis was primarily determined by RA% at 60 min, which showed full concordance with the visual time-activity curve assessment in this cohort; T½ and RA% at 30 min were considered supportive and descriptive parameters, respectively, and did not independently alter the classification. Where two scintigraphies were performed, a patient was classified as having scintigraphic DGE if either examination met the above criteria.

### 2.4. Statistical Analysis

This pilot feasibility study included a small sample size (n = 6); therefore, only descriptive statistics were performed. Continuous variables are reported as median with interquartile range (IQR), and categorical variables as counts and percentages. Analyses were conducted using IBM SPSS Statistics, Version 31 (latest release, June 2025). The primary goal of this analysis was to explore feasibility and generate preliminary data rather than to test formal hypotheses.

## 3. Results

### 3.1. Patient and Preoperative Characteristics

Six patients, who underwent a total of eleven scintigraphic studies, were included in the analysis. The cohort included four females and two males, with ages ranging from 50 to 83 years. Five patients were diagnosed with pancreatic ductal adenocarcinoma (PDAC), while one had a gastrointestinal stromal tumor (GIST). Among the PDAC patients, four underwent upfront surgical resection followed by adjuvant chemotherapy, while one received neoadjuvant chemotherapy prior to surgery. The patient with GIST was treated with neoadjuvant imatinib followed by surgical resection. Performance status, assessed by ECOG criteria, ranged from 1 to 3. Most patients had an ECOG status of 1, indicating they were fully ambulatory, except for the GIST patient and one PDAC patient who had higher scores, reflecting more significant functional impairment. The ASA classification was predominantly class 3, indicating severe systemic disease, with one patient classified as ASA 2. Comorbid conditions such as diabetes mellitus were present in two of the six patients, both diagnosed with PDAC. Interestingly, none of the patients had a history of smoking. Jaundice was present in two PDAC patients, one of whom required preoperative biliary stenting (Table 1).

### 3.2. Intraoperative and Pathological Findings

Five patients underwent a pylorus-preserving pancreaticoduodenectomy (PpPD) while one patient received a pylorus-resecting pancreaticoduodenectomy (PrPD). All patients were operated on via an open approach. Vascular resection was performed in two cases. One patient required venous resection involving mesenteric-splenic venous confluence, while another underwent arterial divestment. Operation times varied considerably, ranging from 211 to 400 min. The majority of tumors were located in the pancreatic head, except for one case involving the duodenum, which corresponded to the patient with a GIST. According to the 8th edition of the TNM classification, all tumors were staged as either pT2 or pT3, with nodal involvement present in five out of six patients. Lymph node positivity ranged from one to fourteen nodes, with total examined nodes varying between 24 and 46. None of the cases showed distant metastasis at the time of surgery. Tumor grading was available for four patients, revealing moderately to poorly differentiated tumors (G2–G3), while grading data was not reported for two patients. All surgical resections achieved negative margins (R0). Features of aggressive tumor biology, including perineural invasion and lymphovascular invasion, were present in four patients with PDAC. No patient showed evidence of microvascular invasion (Table 2).

### 3.3. Postoperative Outcomes

No patient experienced major postoperative complications classified as Clavien-Dindo grade 3 or higher. There were no instances of biliary fistula, abdominal collections, postpancreatectomy hemorrhage (PPH), sepsis, pulmonary complications, or reoperations. All patients survived the postoperative period without in-hospital mortality. Biochemical Leak occurred in only one patient. DGE was identified in two patients, graded as B and C, respectively. Both of these patients required reinsertion of a nasogastric tube, with prolonged nasogastric decompression for 8 and 19 days. These two patients showed markedly delayed tolerance to oral intake. Liquid intake was not tolerated in one of the DGE cases during the recorded postoperative period, while in the other it was initiated on postoperative day 12. Solid food tolerance was similarly delayed, with initiation on postoperative day 14 in one case, while data was not recorded for the other. In contrast, patients without DGE were able to tolerate oral liquids within the first postoperative day and resumed solid intake by days 3 to 5. Length of hospital stay ranged from 11 to 21 days, with longer stays noted in patients with DGE. Intensive care unit (ICU) stays were short, typically between 1 and 3 days. Surgical site infection (SSI) occurred in only one patient during the in-hospital stay, and it did not contribute to prolonged hospitalization or require reoperation (Table 3).

### 3.4. Scintigraphy Analysis (Table 4)

Gastric scintigraphy was performed at two time points for five of six patients, between postoperative days 4–6 (Scintigraphy 1) and 10–13 (Scintigraphy 2), allowing for both early and evolving assessments of gastric function.

**Table 4:** Gastric scintigraphy parameters by patient.

Patients	Date of First Scintigraphy	RA% at 30 min	RA% at 60 min	T 1/2 (min)	Results	Date of Second Scintigraphy	RA% at 30 min	RA% at 60 min	T 1/2 (min)	Overall Scintigraphic Diagnosis
1	04.02.2025	39.3	21.4	43	No-DGE	11.02.2025	0	0	9	No-DGE
2	11.02.2025	52.8	52.8	70	DGE	17.02.2025	75.9	74.5	>1000	DGE
3	30.05.2025	26.9	24.9	56	No-DGE	NP	NP	NP	NP	NP
4	10.03.2025	61.1	59.7	88	DGE	18.03.2025	49.7	44.8	65	DGE
5	04.04.2025	89.7	91.4	199	DGE	10.04.2025	93.4	93.4	>1000	DGE
6	05.05.2025	47.3	47.3	104	DGE	12.05.2025	44.6	52	117	DGE

RA% = Retention Activity Percentage; DGE = Delayed Gastric Emptying; NP = Not Performed.

**Figure 1:**
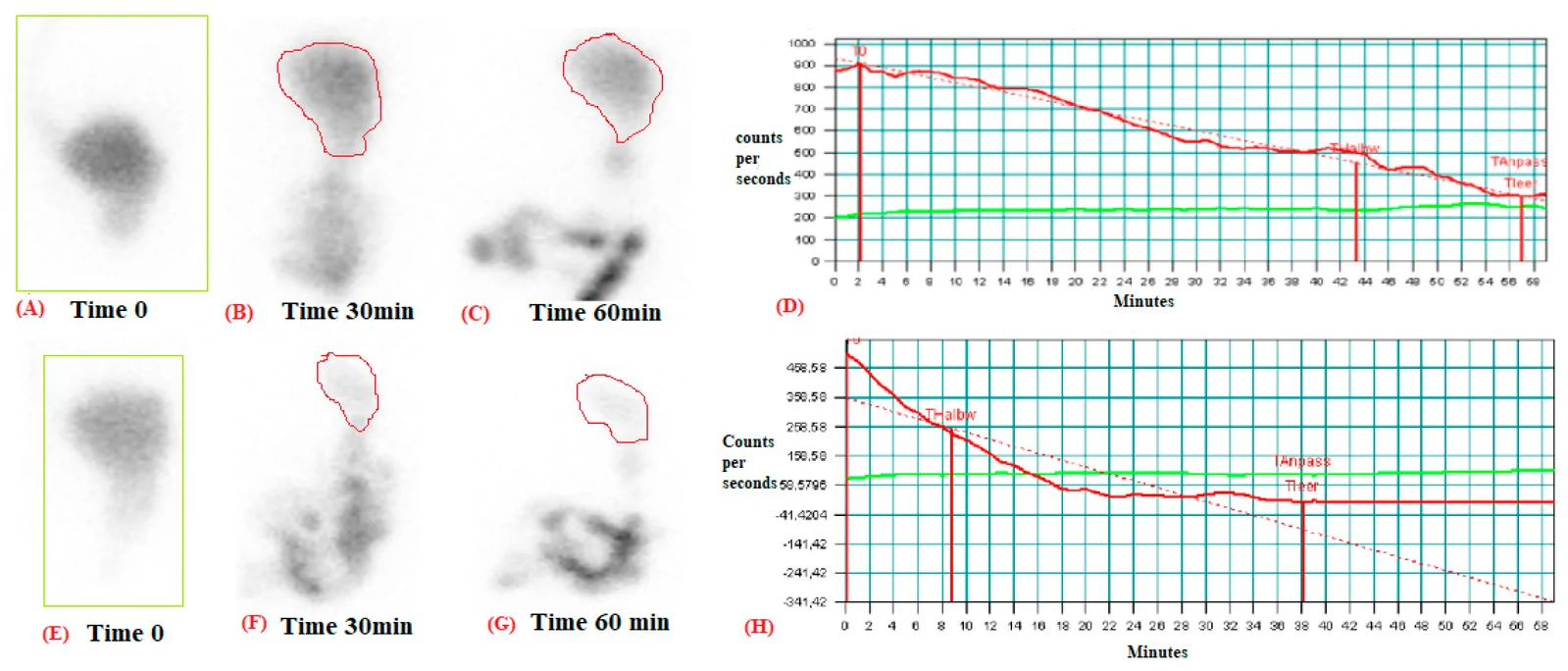
Normal liquid gastric emptying study. Top row, first scintigraphy: Anterior images obtained at 0 min demonstrate a ROI (green) encompassing the entire gastrointestinal activity (**A**), whereas images obtained at 30 and 60 min (**B**,**C**) show a ROI (red) limited to gastric activity. (**D**) Corresponding gastric emptying curve. Bottom row, second scintigraphy (patient 1): Similar findings are demonstrated, with anterior images obtained at 0 min (**E**), 30 min (**F**), and 60 min (**G**), and the corresponding gastric emptying curve (**H**).

Based on T1/2 measurements, two patients (patient 1 and 3) demonstrated normal gastric emptying on scintigraphy. This is exemplified for patient 1 on Figure 1. These two patients concurrently exhibited normal RA% at both 30 and 60 min. In one patient, the second scintigraphy was not performed (patient 3); the other patient showed normal parameters on the second scintigraphy as well, paralleling an uncomplicated postoperative course.

**Figure 2:**
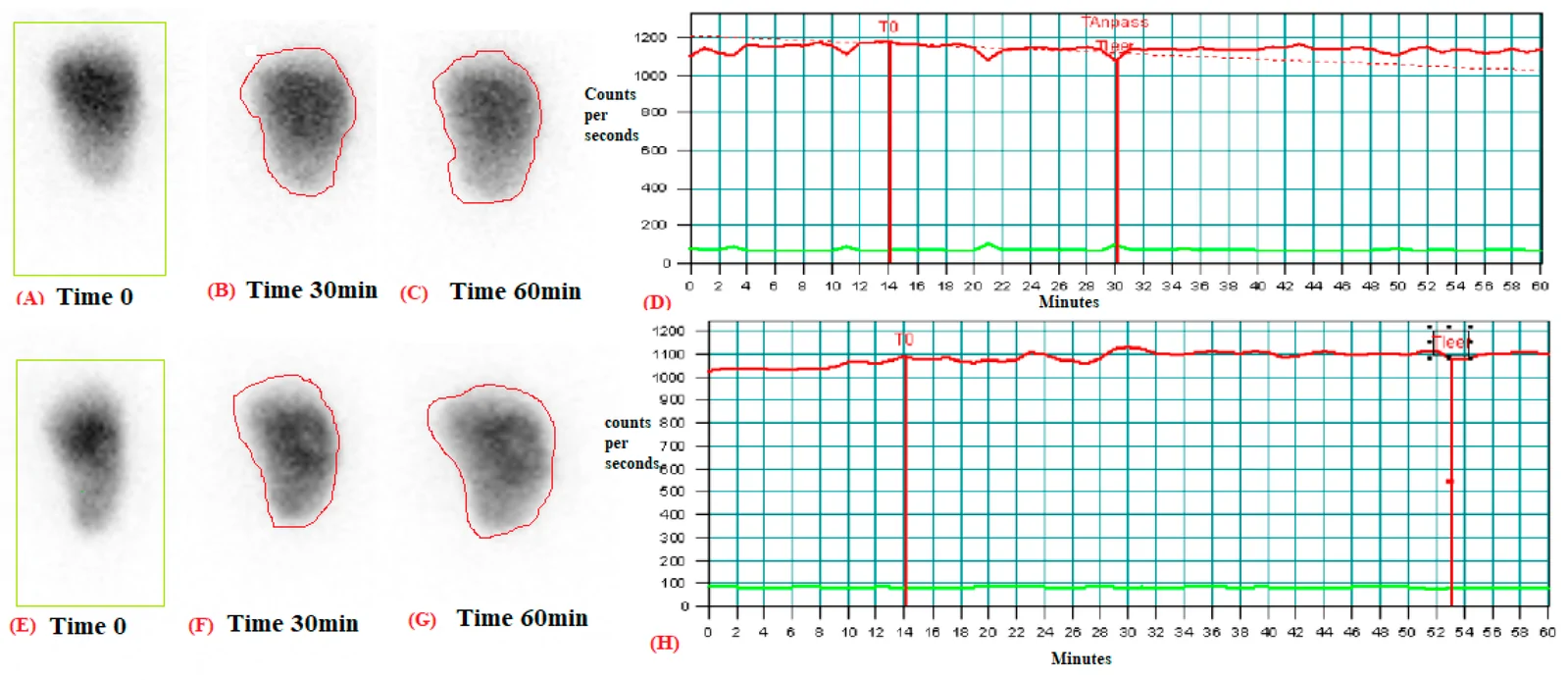
Anterior images obtained at 0, 30, and 60 min demonstrate no radiotracer transit into the bowel, with persistent gastric retention. (**A**–**C**) The corresponding gastric emptying curve is flat, indicating markedly delayed emptying (**D**). Bottom row, (**E**–**H**): Similar findings are observed on the second scintigraphy study (patient 5).

Four of six patients were classified as DGE (Figure 2, patient 5). All these patients demonstrated abnormal T1/2 values on first and RA% above the normal range at 60 min on both the first and second scintigraphy.

Two patients demonstrated persistently elevated RA% at 60 min and markedly prolonged T1/2 on both examinations, accompanied by delayed oral intake and prolonged nasogastric tube requirement (patient 4 and 5) This corresponds to clinically relevant complications according to the ISGPS definition (Grade B/C), although these do not necessarily represent major complications according to the Clavien–Dindo classification.

In contrast, two additional patients showed abnormal scintigraphic parameters, particularly prolonged T1/2 and elevated RA% at 60 min, without fulfilling clinical ISGPS DGE criteria, raising the possibility of subclinical gastric dysmotility; however, no outcome data (e.g., weight loss, quality of life) were available to confirm the clinical significance of this finding (patient 2 and 6).

## 4. Discussion

This pilot feasibility study demonstrates the potential of gastric emptying scintigraphy as an objective tool to evaluate DGE after pancreatoduodenectomy. The current ISGPS definition allows retrospective classification but does not provide guidance for precise diagnosis or management or for quantification in clinical studies. In our series, all patients tolerated the examination showing no complications, supporting its safety and applicability in the postoperative setting. These findings provide the basis for a standardized protocol that can be used in future studies, including predefined time points of measurement, harmonized imaging parameters, and uniform criteria for quantitative assessment. This could enable reproducible evaluation of gastric emptying, facilitate comparison across centers, and support the integration of scintigraphy into clinical trials targeting DGE. This approach aligns with previous single-center series and systematic reviews that highlighted scintigraphy as the most promising modality for objective assessment, despite variability in protocols and limited adoption to date [5,6,7].

In this study, we employed a standardized liquid gastric scintigraphy protocol to evaluate postoperative gastric function. Visual and quantitative assessment of gastric emptying through ROI time-activity curves as well as gastric T1/2 and RA% at 30, and 60 min are provided. It has to be noted that in contrast to solid gastric scintigraphy, there are only limited data on liquid gastric scintigraphy for the diagnosis of DGE [8].

Compared to previous studies that primarily focused on pre- and postoperative gastric scintigraphy to correlate findings with clinically diagnosed DGE, our study employed a more targeted postoperative approach. Specifically, we performed two standardized gastric scintigraphies postoperatively, in the early and the later recovery phase. In contrast, other studies incorporated preoperative scans and assessed patients over longer intervals (e.g., days 10 and 21 post-op), often emphasizing sensitivity and specificity in relation to clinical symptoms rather than detailed kinetic parameters. Our focus on early postoperative gastric kinetics offers a more immediate functional assessment and may enhance early detection of DGE, complementing the more symptom-correlated, time-staggered designs used in other studies [5,6,7].

In our cohort of six patients, 11 postoperative gastric scintigraphies were performed, revealing varying degrees of DGE. Clinically relevant DGE (Grades B and C) was observed in two patients and was consistently associated with delayed gastric emptying on scintigraphy, high RA%, and prolonged T1/2. These findings align with previous studies reporting a strong correlation between high RA at 1 h (e.g., >94% at POD 7) and clinically significant DGE. While our sample size is limited, the scintigraphic pattern, particularly the persistent tracer retention and flat time-activity curves, support the utility of postoperative scintigraphy as an objective tool for detecting and grading DGE [5,6,7]. Additionally, we can conclude that both T1/2 values and RA% at 60 min obtained from first and second scintigraphy demonstrated better concordance with visual interpretation and clinical assessment. However, abnormal RA% at 30 min on the first scintigraphy were observed in patients with severe clinical DGE grading, suggesting that early RA measurements may serve as a potential severity marker in future studies.

Beyond the pathophysiological focus of the present pilot study, several broader aspects of perioperative care for pancreatic head resections merit consideration when interpreting our results. Pancreatoduodenectomy remains a technically demanding procedure with substantial postoperative morbidity, and continued efforts to optimize perioperative outcomes are a central theme in the pancreatic surgery literature [9,10]. System-level factors such as failure to rescue after major complications [11] and centralization of complex pancreatic resections to high-volume centers [12], including the introduction of statutory minimum caseload requirements for pancreatic surgery in Germany [13], likely influence how complications such as DGE are recognized and managed. Multidisciplinary tumor board discussion, performed for all patients in our cohort, is regarded as standard practice for resectability assessment and treatment allocation in pancreatic cancer [14]. Surgical technique may also play a role: early experience with robotic pancreatoduodenectomy suggests outcomes comparable to the open approach [15], and prehabilitation programs before pancreatic cancer surgery have been proposed to improve functional recovery, although adherence to home-based exercise protocols remains variable [16,17]. Artificial intelligence-assisted image guidance is increasingly explored as an adjunct in minimally invasive pancreatic surgery [18], and risk-adapted analysis of complex oncological resections involving the pancreas, as we have described elsewhere, may help contextualize complication profiles such as DGE within a broader case-mix [19]. In this regard, a predefined analysis of the randomized DIPLOMA-2 trial recently found comparable postoperative activity levels and physiological stress after minimally invasive and open pancreatoduodenectomy [20], in line with a meta-analysis of randomized trials comparing minimally invasive and open pancreatic surgery more broadly [21]. Our institutional approach follows the current German national guideline for exocrine pancreatic cancer [22], within which standardized, objective assessment tools such as gastric scintigraphy could eventually be incorporated.

This study has important limitations, including its very small sample size, and the single-center setting, which restrict generalizability. Further, patients were selected retrospectively and non-consecutively based on clinical indication for scintigraphy rather than as a predefined, consecutive cohort, introducing selection bias. The normal reference values used to define delayed gastric emptying were derived from a non-postoperative population [8], and their applicability to the early postoperative setting has not been independently validated. Preoperative gastric scintigraphy was not performed, so individual baseline gastric motility could not be accounted for. Only one patient developed a Biochemical Leak, precluding any meaningful assessment of the relationship between POPF and DGE in this cohort. Finally, no longitudinal outcome data (e.g., weight loss, nutritional status, quality of life, or delayed adjuvant therapy) were collected, so the clinical significance of scintigraphic abnormalities in patients without ISGPS-defined DGE (“subclinical” dysmotility) remains unconfirmed and should be interpreted as hypothesis-generating rather than established. Nevertheless, we were able to demonstrate the feasibility of performing gastric emptying scintigraphy early after pancreatoduodenectomy, with all patients tolerating the examination without complications. Importantly, we also outlined a standardized protocol that can serve as a foundation for future prospective studies. Such studies should evaluate interventions targeting DGE using reproducible, objective endpoints, thereby advancing both clinical management and the design of interventional trials. From a clinical research perspective, the ability of scintigraphy to detect subclinical gastric emptying delay may have important implications for endpoint definition in future trials. Reliance solely on clinical criteria, such as nasogastric tube reinsertion or delayed oral intake, may underestimate the true incidence of postoperative gastric dysmotility. Importantly, we showed concordant findings in clinically relevant ISGPS Grade B/C DGE and additionally identified subclinical gastric emptying delay in patients without overt clinical DGE, suggesting higher sensitivity compared to clinical criteria alone. Therefore, incorporating scintigraphic parameters into clinical trial design could enable a more precise and pathophysiology-based definition of DGE, improve risk stratification, and enhance the sensitivity of outcome assessment. Such an approach may be particularly valuable in interventional studies evaluating surgical techniques, reconstruction methods, or perioperative management strategies after pancreaticoduodenectomy.

## 5. Conclusions

Standardized gastric scintigraphy may serve as a practical and reproducible tool for the objective evaluation of DGE after pancreaticoduodenectomy. Beyond its diagnostic value, scintigraphy offers quantitative and observer-independent parameters (T1/2, RA% at defined time points) that may complement current clinically based definitions such as ISGPS criteria. A central question, however, remains the definition of an appropriate reference standard. Clinical DGE grading relies on surrogate markers, nasogastric tube reinsertion, delayed oral intake, and length of stay, which reflect consequences of impaired gastric emptying rather than gastric physiology itself. Scintigraphy, in contrast, directly measures functional gastric transit. Therefore, future studies should clarify whether scintigraphic findings correlate consistently with clinical outcomes, other functional parameters, and longitudinal patient follow-up. Systematic postoperative follow-up would be essential to determine whether subclinical scintigraphic abnormalities translate into prolonged recovery, reduced quality of life, nutritional impairment, or delayed adjuvant therapy, thereby defining the true clinical relevance of scintigraphy-detected DGE.

## Figures and Tables

**Table 1:** Patient and preoperative characteristics.

Case ID	1	2	3	4	5	6
Age (years)	56	83	64	50	83	62
Sex (m/f)	f	f	f	m	m	f
Diagnosis	PDAC	PDAC	PDAC	GIST	PDAC	PDAC
Treatment Strategy	Upfront resection and adjuvant chemotherapy	Upfront resection and adjuvant chemotherapy	Upfront resection and adjuvant chemotherapy	Neoadjuvant Imatinib and surgical resection	Upfront resection and adjuvant chemotherapy	Neoadjuvant chemotherapy and surgical resection
ECOG Status	1	1	1	3	1	2
ASA Classification	2	3	3	3	3	3
History of Diabetes Mellitus	No	No	Yes	No	Yes	No
History of Smoking	No	No	No	No	No	No
Jaundice	Yes	No	Yes	No	No	No
Preoperative Biliary Stent	No	No	Yes	No	No	No

**Table 2:** Intraoperative and pathological findings.

Case ID	1	2	3	4	5	6
Surgical Procedure	PpPD (Open)	PpPD (Open)	PpPD (Open)	PpPD (Open)	PpPD (Open)	PrPD
Vascular Resection	Yes	No	No	No	No	Yes
Type of Vascular Resection	VMS	No	No	No	No	Arterial Divestment
Operation Time (min)	269	306	238	363	211	400
Tumor Location	Pancreatic head	Pancreatic head	Pancreatic head	Duodenum	Pancreatic head	Pancreatic head
Histology	PDAC	PDAC	PDAC	GIST	PDAC	PDAC
(y)pT Stage (TNM 8th edition)	3	2	2	2	2	2
(y)pN Stage (TNM 8th edition)	2	1	1	0	2	1
Number Lymph Nodes (Positive/Examined)	14/46	1/34	1/24	0/25	3/26	3/27
(y)cMetastasis (TNM 8th edition)	No	No	No	No	No	No
Grading (G)	2	3	3	-	2	-
Resection Margin (R)	0	0	0	0	0	0
Perineural Invasion	1	1	1	0	1	0
Lymphovascular Invasion	1	1	1	0	1	0
Microvascular Invasion	0	0	0	0	0	0

**Table 3:** Postoperative outcomes.

Case ID	1	2	3	4	5	6
Morbidity ≥ 3 Clavien Dindo	No	No	No	No	No	No
Biochemical Leak	Yes	No	No	No	No	No
POPF (Grade B)	No	No	No	No	No	No
POPF (Grade C)	No	No	No	No	No	No
Biliary Fistula	No	No	No	No	No	No
Abdominal Collection	No	No	No	No	No	No
DGE Grade	No	No	No	C	B	No
Nasogastric tube reinsertion	No	No	No	Yes	Yes	No
Duration of nasogastric tube	0	0	0	19	8	0
Postoperative day of tolerated oral liquid intake	1	1	1	-	12	1
Postoperative day of tolerated oral solid intake	3	5	4	-	14	5
PPH	No	No	No	No	No	No
Sepsis	No	No	No	No	No	No
Pulmonary Complications	No	No	No	No	No	No
Length of Stay (days)	19	14	11	21	19	13
Length of ICU Stay (days)	3	2	1	1	1	1
Reoperation	No	No	No	No	No	No
In-hospital Mortality	No	No	No	No	No	No
Postoperative SSI in-hospital	Yes	No	No	No	No	No

## Data Availability

The data presented in this study are available on request from the corresponding author due to institutional data protection restrictions on patient-level clinical data.
